# Thrombocytopenia in the first trimester predicts adverse pregnancy outcomes in obstetric antiphospholipid syndrome

**DOI:** 10.3389/fimmu.2022.971005

**Published:** 2022-08-18

**Authors:** Jiayang Jin, Xue Xu, Lei Hou, Yuke Hou, Jing Li, Meiying Liang, Chun Li

**Affiliations:** ^1^ Department of Rheumatology and Immunology, Peking University People’s Hospital, Beijing, China; ^2^ Beijing Key Laboratory for Rheumatism Mechanism and Immune Diagnosis (BZ0135), Beijing, China; ^3^ Obstetrics and Gynecology Department, Peking University People’s Hospital, Beijing, China; ^4^ The Second Affiliated Hospital of Guizhou University of Traditional Chinese Medicine, Guiyang, China

**Keywords:** antiphospholipid syndrome, preterm birth, small-for-gestational age, thrombocytopenia, intrauterine fetal death

## Abstract

**Background:**

Thrombocytopenia is a common manifestation of antiphospholipid syndrome (APS), and is a main concern for bleeding on the standard treatment of low dose aspirin (LDA) and low molecular weight heparin (LMWH) in obstetric APS (OAPS).

**Objective:**

This study assesses the possible relationship between thrombocytopenia during the first trimester and adverse pregnancy outcomes (APOs) in OAPS patients.

**Methods:**

A case-control study was conducted at Peking University People’s Hospital, Beijing, China. The clinical, immunologic, and pregnancy outcomes of the OAPS patients were collected. Univariate and multivariate logistic regression analyses were applied to assess the relationship between APOs and thrombocytopenia in the first trimester.

**Results:**

A total of 115 participants were included in the analysis. There were no difference on antepartum and postpartum hemorrhage between the two groups. The gestational age in the thrombocytopenia group was less than that in the control group (34.12 ± 8.44 vs. 37.44 ± 3.81 weeks, *p* = 0.002). Hypocomplementemia, double aPL positive, and high titers of anti-β2 glycoprotein I were more frequent in APS patients with thrombocytopenia (*p* < 0.05). Compared to the control group, thrombocytopenia in the first trimester was correlated with SGA (12.12% vs. 31.25%, *p* = 0.043), premature birth <37 weeks (16.16% vs 43.75%, *p* = 0.010) and intrauterine fetal death (2.02% vs 12.50%, *p* = 0.033). Thrombocytopenia in first-trimester independently increased the risk of preterm birth <37 weeks (OR = 5.40, 95% CI: 1.35-21.53, *p *= 0.02) after adjusting for demographic and laboratory factors. After adding medication adjustments, these factors above become insignificant (*p* > 0.05). Of note, the number of platelets increased after delivery in 14 thrombocytopenia patients with live fetuses (*p* = 0.03).

**Conclusion:**

This study demonstrates that thrombocytopenia in the first trimester increases the risks of preterm birth in women with APS. The effective OAPS treatments may improve pregnancy outcomes and not increase the risk of antepartum and postpartum hemorrhage.

## Introduction

Antiphospholipid syndrome (APS) is described as an autoimmune condition characterized by arterial or venous thrombosis and/or pregnancy complications, accompanied by persistent antiphospholipid antibodies (aPLs) (1, 2). The classical aPLs are anticardiolipin antibodies (aCL), anti-β2 glycoprotein I (aβ2GPI), and lupus anticoagulant (LA) (3, 4). Platelet activation is one stage of etiopathogenesis (4), while the binding of aPLs to β2GPI on platelets leads to its activation and aggregation (5).

Thrombocytopenia is a common hematologic manifestation in APS patients, with a prevalence ranging from 16 to 53% (6). In several studies, the frequencies of thrombocytopenia were higher than pregnancy morbidity (7, 8). Differences in prevalence partially depend on different threshold descriptions of thrombocytopenia (<100×10^9^/L or <150×10^9^/L). In a prospective study with individuals with platelet counts between 100 and 150×10^9^/L, 88% of these platelet counts reached normal values or remained stable without any treatment during follow-up (9).

Several studies have investigated the relationship between thrombocytopenia and clinical APS features. Patients with thrombocytopenia had a higher incidence of cardiac valve thickening and dysfunction, epilepsy, chorea, arthritis, livedo reticularis, and skin ulcerations (10). However, another study did not find any correlations between thrombocytopenia and clinical manifestations in APS (11). The presence of thrombocytopenia could be a marker for high-risk APS patients (12).There have been few reports assessing the relationship between thrombocytopenia and pregnancy complications in APS patients.

The platelet counts decrease during normal pregnancy and increase postpartum (13). The timing of thrombocytopenia is also an important differentiate character from hypertension or gestational thrombocytopenia (14). Given the absence of management guidelines for immune thrombocytopenia in obstetric APS(OAPS), treatment recommendations for severe thrombocytopenia have typically been adapted from ITP (15). However, OAPS differ from ITP since these patients have hypercoagulability with thrombocytopenia. Low-dose aspirin (LDA) and low molecular weight heparin (LMWH) are currently accepted first-line treatments for OAPS (16), which are not included in ITP management. However, the treatment of LDA and LMWH also have the potential risk of thrombocytopenia. Therefore, physicians may avoid prescribing LDA and LMWH for fear of bleeding. Considering the high prevalence of low platelet and treatment dilemmas in OAPS, we sought to evaluate possible associations between thrombocytopenia and adverse pregnancy outcomes (APOs) in OAPS patients. What’s more, thrombocytopenia throughout pregnancy cannot exclude gestational hypertension and physiological thrombocytopenia in the second and third trimester of pregnancy. The relationship between thrombocytopenia in the first trimester and APOs in APS patients is worth to explore.

## Materials and methods

### Study population

Data from all OAPS patients were retrospectively reviewed between January 2013 and June 2021 at Peking University People’s Hospital. All patients fulfilled the Sydney classification criteria of APS (17). Thrombocytopenia was defined as platelet levels less than 100 ×10^9^/L.

The inclusion criteria were: 1) live fetus diagnosed by ultrasonic examination at 6 weeks; 2) diagnosis of primary APS before or during pregnancy; 3) complete clinical and follow-up data. The exclusion criteria were: 1) voluntary interruption of pregnancy; and 2) thrombocytopenia caused by other causes, including hematological diseases, EDTA-dependent pseudo thrombocytopenia, and splenomegaly; 3) patients whose clinical data were missing, as well as those who were lost during follow-up.

This study was conducted in accordance with the Declaration of Helsinki and was approved by the ethics committee of Peking University People’s Hospital (2019PHB252). All patients signed written informed consents before participating in this study.

### Data collection and follow-up

Demographic and clinical data included age, gestational age, obstetric history, previous abortion history (early abortion<10 weeks, late abortion≥10 weeks), previous thrombosis, pregnancy, and fetal outcomes.

Laboratory data included antinuclear antibody (ANA), hypocomplementemia (low complement C3/C4), aCL, aβ2GPI, and LA. Treatment included prednisone (pred), hydroxychloroquine (HCQ), LMWH, LDA, intravenous immunoglobulin (IVIg), and calcineurin inhibitors.

### Adverse pregnancy outcomes

Adverse pregnancy outcomes (APOs) were classified as follows: preeclampsia (18, 19); preterm birth <37 weeks gestational age excluding excessive membrane stretching, prior cervical surgery and choriodecidual infection (20); premature rupture of membranes (21); small-for-gestational-age (SGA) defined as birthweight below the 10th percentile (22–25); stillbirth defined as fetal death after 20 weeks (26); intrauterine fetal death defined as fetal death before 20 weeks (27).

### Statistical analysis

Categorized variables are shown as a frequency or percentage, and continuous variables are shown as mean ± standard deviation (SD). The Mann-Whitney and Chi-square tests were used to determine any statistical difference between the means and proportions of the two groups. Both non-adjusted and multivariate-adjusted models were applied. Univariate and multivariate logistic regression analyses were applied to assess the relationship between APOs and thrombocytopenia. All analyses were performed with the statistical software packages R version 3.4.3 (http://www.R-project.org, The R Foundation) and EmpowerStats (http://www.empowerstats.com, X&Y Solutions, Inc., Boston, MA). A two-sided significance of 0.05 was considered statistically significant.

## Results

### Clinical characteristics of APS patients with or without thrombocytopenia in the first trimester

A total of 124 pregnant women were screened for in this study, and 9 participants were excluded because they did not meet the inclusion criteria. The remaining 115 patients were divided into the control group (243.52 ± 66.88×10^9^/L, N = 99) and thrombocytopenia in the first-trimester group (54.06 ± 22.46×10^9^/L, N = 16) according to the number of platelets. The platelet count of 6 participants was lower than 50×10^9^/L (**Table 1**). Compared with the control group, the thrombocytopenia group had a lower previous abortion rate (77.78% vs. 37.50%, *p* = 0.001), especially lower early abortion rate (62.63% vs. 25.0%, *p* = 0.006). In the thrombocytopenia group, there was a lower rate of APS diagnosis before pregnancy (37.5% vs. 87.88%, *p* < 0.001).

**Table 1:** Basic and gestational characteristics between the two groups.

	Thrombocytopenia group (N = 16)	Control group (N = 99)	*p*-value
Platelet counts (×10^9^/L)	54.06 ± 22.46	243.52 ± 66.88	<0.001*
Platelet counts ≤ 50×10^9^/L, n (%)	6 (37.50)	0 (0.00)	<0.001*
Age, years	31.5 ± 4.1	32.8 ± 3.8	0.289
Age ≥ 35 years, n (%)	2 (12.50)	20(20.20)	0.467
Previous abortion, n (%)	6 (37.50)	77 (77.78)	0.001*
Early abortion, n (%)	4 (25.00)	62 (62.63)	0.006*
Late abortion, n (%)	2 (12.50)	32 (32.32)	0.112
History of thrombosis, n (%)	0 (0.00)	5 (5.05)	0.361
Nulliparity, n (%)	14 (87.50)	71 (71.72)	0.182
Diagnosis of APS before pregnancy, n (%)	6 (37.50)	87 (87.88)	<0.001*
Gestational age, weeks	34.12 ± 8.44	37.44 ± 3.81	0.023*
Full term birth, n (%)	9 (56.25)	85 (85.86)	0.006*
Pregnancy by artificial insemination, n(%)*	0 (0.00)	21 (21.21)	0.043*
Live fetus, n (%)	14 (87.50)	96(96.97)	0.142
antepartum hemorrhage, n (%)	7 (43.75)	27 (27.27)	0.180
Postpartum hemorrhage, n (%)	5 (33.33)	21 (21.65)	0.319
Amount of bleeding(ml)	436.67 ± 316.49	320.10 ± 244.91	0.176
ANA positive, n (%)	5 (35.71)	21 (25.61)	0.432
ANA titer, n (%)			0.525
1:40	1 (7.14)	11(13.41)	
1:80	2 (14.29)	4 (4.88)	
1:160	1 (7.14)	4 (4.88)	
1:320	1 (7.14)	2 (2.44)	
Hypocomplementemia, n (%)	6 (37.50)	16 (16.16)	0.042*
High titer of aβ2GPI and/or aCL, n (%)	11 (68.75)	27 (27.27)	<0.001*
LA positive, n (%)	9 (56.25)	22 (22.22)	0.004*
Double aPL positive, n (%)	9 (56.25)	25 (25.25)	0.011*
Triple aPL positive, n (%)	4 (25.00)	9 (9.09)	0.060
Anti-dsDNA titer, IU/ml	7.69 ± 7.06	6.85 ± 5.87	0.808
Anti-dsDNA positive, n (%)	0 (0.00%)	1 (1.01%)	0.999
Without Treatment, n (%)	1 (6.25)	2 (2.02)	0.320
Pred, mg/d	13.75 (7.50-16.25)	0.00 (0.00-0.00)	<0.001*
Pred, n (%)	12 (75.00)	24 (24.24)	<0.001*
HCQ, mg/d	400.00 (200.00-400.00)	400.00 (0.00-400.00)	0.470
HCQ, n (%)	13 (81.25)	68 (68.69)	0.284
LMWH, IU/d	0.00 (0.00-1025.00)	4100.00 (4100.00-4100.00)	<0.001*
LMWH, n (%)	4 (25.00)	79 (79.80)	<0.001*
Aspirin, mg/d	0.00(0.00-12.50)	75(0.00-100.00)	<0.001*
Aspirin, n (%)	4 (25.00)	72 (72.73)	<0.001*
Aspirin+ LMWH, n (%)	1 (6.25)	56 (56.57)	<0.001*
IVIg, n (%)	4 (25.00)	2 (2.02)	<0.001*

*P < 0.05. APS, antiphospholipid syndrome; ANA, antinuclear antibody (ANA); hypocomplementemia, low C3/C4: complement C3/C4; aPLs: antiphospholipid antibodies included cardiolipin antibodies (aCL), anti-β2 glycoprotein I (aβ2GPI), and lupus anticoagulant (LA); Pred, prednisone; HCQ, hydroxychloroquine; LMWH: low molecular weight heparin, IVIg, Intravenous Immunoglobulin Therapy.

The percentage of pregnancy by artificial insemination in the control group was higher than in the thrombocytopenia group (21.21% vs. 0.00%, *p* = 0.043). Gestational age in the thrombocytopenia group was less than that in the control group (34.12 ± 8.44 vs. 37.44 ± 3.81 weeks, *p* = 0.0023) (**Table 1**). Hypocomplementemia, single aPL positive, high titer of aβ2GPI and/or aCL, and LA positive were all more frequent in APS patients with thrombocytopenia in the first trimester (*p*<0.05) (**Table 1**). The prevalence of double aPL positive was higher in the thrombocytopenia group than in the control group (56.25% vs. 25.25%, *p*=0.011) (**Table 1**). There was no difference of antepartum (27.27% vs. 43.75%, *p*= 0.180) and postpartum (21.65% vs. 33.33%, *p* = 0.319) hemorrhage between the two groups.

The dosage and utilization rate of prednisone was higher in the thrombocytopenia group (75.00% of patients with a median dose of 13.75 mg/day) than in the control group (24.24% of patients with a median dose of 0.00 mg/day). The thrombocytopenia group was treated less frequently with LMWH (25.00% vs. 79.80%), aspirin (25.00% vs. 72.73%), and LMWH+aspirin (6.25% vs. 56.57%) than the control group (All *p* < 0.001). Additionally, therapeutic doses of LMWH and aspirin in the control group [4100.00 (4100.00-4100.00) IU/day and 75(0.00-100.00) mg/day, respectively] were significantly higher than in the thrombocytopenia group [0.00 (0.00-1025.00) IU/day and 0.00 (0.00-12.50) mg/day, respectively] (All *p* < 0.001). The use of IVIg in the thrombocytopenia group was higher than in the control group (25.00% vs. 2.02%, *p* < 0.001). (**Table 1**).

### Relationship between thrombocytopenia in the first trimester and APOs

Among patients with thrombocytopenia in the first trimester, 31.25% patients (N=5) were combined with SGA, while the proportion was only 12% (N=12) in the control group (*p* = 0.043). Compared to the control group, thrombocytopenia in the first trimester was correlated with premature birth <37 weeks (16.16% vs 43.75%, *p* = 0.010) and intrauterine fetal death (2.02% vs 12.50%, *p* = 0.033) (**Table 2**). In the thrombocytopenia group, patients who were diagnosed with low platelet count during pregnancy have a higher rate of intrauterine fetal death compared to the patients who were known to have low platelet count (100% vs 0.00%, *p *= 0.008) (**Supplementary Table 1**).

**Table 2:** Adverse pregnancy outcomes in the thrombocytopenia in the first-trimester group and control group.

	Thrombocytopenia group (N = 16)	Control group (N = 99)	*p*-value
SGA, n (%)	5 (31.25)	12 (12.12)	0.043*
Preeclampsia n (%)	1 (6.25)	9 (9.19)	0.716
Premature birth <37 weeks, n (%)	7 (43.75)	16 (16.16)	0.010*
Stillbirth, n (%)	0 (0.00)	2 (2.02)	0.568
Intrauterine fetal death, n (%)	2 (12.50)	2 (2.02)	0.033*
PROM, n (%)	1 (6.25)	25 (25.25)	0.095

*P < 0.05. SGA, small for gestational age; PROM, Premature rupture of membranes.

Univariate logistic regression analysis (**Table 3**) indicated that thrombocytopenia in the first trimester was associated with premature birth < 37 weeks (OR = 4.08, 95%CI: 1.33-12.55, *p* = 0.01). Thrombocytopenia in the first trimester had an increased risk of APOs, including SGA (OR=3.33, 95%CI: 0.99-11.26, *p* = 0.05) and intrauterine fetal death (OR=7.00, 95%CI: 0.91-53.75, *p* = 0.06).

**Table 3:** Univariate and multivariate logistic regression analyses of thrombocytopenia in first-trimester associated with adverse pregnancy outcomes in patients with APS.

Thrombocytopenia in first-trimester	Unadjusted	Model I	Model II
SGA	3.33 (0.99, 11.26) *p* = 0.05	2.48 (0.64, 9.58) *p* = 0.18	3.68 (0.81, 16.77) *p* = 0.09
Premature birth <37 weeks	4.08 (1.33, 12.55) *p* = 0.01	5.22 (1.43, 19.07) *p* = 0.01	5.40 (1.35, 21.53) *p* = 0.02
Intrauterine fetal death	7.00 (0.91, 53.75) *p* =0.06	NA	NA

SGA, small for gestational age.

Adjust I model adjust for: age; previous abortion history; pregnancy by artificial insemination

Adjust II model adjust for: age; previous abortion history; pregnancy by artificial insemination. hypocomplementemia; high titer of aβ2GPI and/or aCL. NA, Not Applicable.

The multivariate analysis of the relationship between thrombocytopenia in the first trimester and APOs are presented in **Table 3**. Thrombocytopenia in the first trimester independently increased the risk of premature birth <37 weeks (Model I: OR = 5.22, 95% CI: 1.43-19.07, *p* = 0.01; Model II: OR = 5.40, 95% CI: 1.35-21.53, *p* = 0.02). In the adjusted models I and II, the relationship between thrombocytopenia in the first trimester and SGA were not significant (*P >*0.05) compared with the control group.

After adding the adjustments of therapeutic medication such as prednisone, HCQ, IVIg, LDA, and LMWH, with single or combined usage, the influences on APOs mentioned above become insignificant, which indicates that treatment could decrease the risk of APOs with thrombocytopenia (*p* > 0.05 as shown in **Table 4**).

**Table 4:** Medication treatment changed the adverse pregnancy outcomes in APS patients with thrombocytopenia in first-trimester.

Thrombocytopenia in first-trimester	Model I	Model II	Model III
SGA	3.70 (0.76, 18.05) *p* = 0.11	1.84 (0.32, 10.62) *p* = 0.49	0.67 (0.07, 6.64) *p* = 0.73
Premature birth <37 weeks	4.75 (1.13, 20.03) *p* = 0.03	3.17 (0.66, 15.28) *p* = 0.15	2.13 (0.34, 13.34) *p* = 0.42
Intrauterine fetal death	NA	NA	NA

SGA, small for gestational age.

Adjust I model adjust for: age; previous abortion history; pregnancy by artificial insemination; hypocomplementemia; High titer of 2gp1 and/or ACL; aspirin+low molecular weight heparin.

Adjust II model adjust for: age; previous abortion history; pregnancy by artificial insemination; hypocomplementemia; high titer of aβ2GPI and/or aCL; pred; IVIg.

Adjust III model adjust for: age; previous abortion history; pregnancy by artificial insemination; hypocomplementemia; high titer of aβ2GPI and/or aCL; pred; HCQ; aspirin; low molecular weight heparin; aspirin+low molecular weight heparin; IVIg. NA, Not Applicable.

### Changes in platelets at different trimesters of pregnancy in thrombocytopenia patients

The following analysis compared the number of platelets in different pregnancy trimesters of 14 thrombocytopenia patients with a live fetus, including the first trimester, the second trimester, the third trimester, and 42 days postpartum (**Supplementary Figure 1**). The results indicate that the number of platelets increased after delivery with the treatment (57.64 ± 5.77 vs. 86.79 ± 9.65×10^9^/L, *p* = 0.03).

## Discussion

Our study found that the prevalence of thrombocytopenia in the first trimester was 13.8% in OAPS patients. The rate of thrombocytopenia in our study was slightly lower than in a previous study (6), which could be due to differences in our defined platelet threshold and thrombocytopenia we counted in the first trimester not the whole pregnancy.

Several studies reported that the mechanism of thrombocytopenia in APS could be platelet consumption and/or destruction mediated by aPLs (28, 29). Platelets could also play a central role in APS as the main target of β2GPI (30, 31). Therefore, thrombocytopenia could be a predictive factor of clinical manifestations, including obstetrical morbidity in APS (32–34). In our study, hypocomplementemia and single/double-positive aPLs were found to be more frequent in APS patients with thrombocytopenia in the first trimester, which could indicate the pathogenic effect of antiphospholipid antibodies on the platelets.

Many studies reported that previous thrombosis, triple aPLs positivity, and LA positive were the factors related to various APOs (35–37). Little research reported the association between thrombocytopenia in the first trimester and pregnancy outcomes. Our study demonstrated that thrombocytopenia in the first trimester was correlated with SGA, premature birth <37 weeks and intrauterine fetal death compared to the control group. What’s more, thrombocytopenia in the first trimester independently increased the risk of premature birth <37 weeks after adjustment for demographic and immunologic confounders. A recent study showed that rates of intrauterine growth retardation, recurrent fetal loss, and pregnancy toxemia were similar in APS patients with and without thrombocytopenia (10). These results differ from previous studies, which could be due to the study population and the definition of APOs. More importantly, the factors causing this difference may be thrombocytopenia in previous papers cannot completely exclude pregnancy induced hypertension or thrombocytopenia caused by gestational thrombocytopenia in the second and third trimester. Thrombocytopenia during the first trimester, which is usually the first time many patients receive a blood test, is an indicator for APOs in APS patients.

Our results showed the therapeutic dose of LMWH and aspirin in the thrombocytopenia group is lower than the dose in the control group, which is in line with the guidelines. Aspirin and LMWH did not increase the risk of poor pregnancy outcomes as previously reported (5). Additionally, in our study, anti-coagulation therapy did not increase the incidence of antepartum bleeding in patients with thrombocytopenia. The data showed that after adjusting the treatment factors, the prognosis of APS patients with thrombocytopenia could be improved. It is recommended that full-dose enoxaparin be provided for patients with platelet counts greater than 50×10^9^/L and half-dose enoxaparin for platelet counts between 25×10^9^/L and 50×10^9^/L (38). These findings indicate that pregnant APS patients with thrombocytopenia in the first trimester should receive positive treatment.

Our findings showed that the dosage and utilization rate of prednisone was much higher in the thrombocytopenia group. Glucocorticoids are the first-line treatment for APS-associated immune thrombocytopenia (ITP) (15, 39). For pregnant women with ITP, the optimal dose of corticosteroids has not been determined. It is reported that treatment with corticosteroids at doses ≥15 mg/day during pregnancy and delivery is correlated with a higher incidence of complications in ITP mothers and infants, including premature labor and preeclampsia in the mother and abnormal body weight and congenital abnormalities in the infant (40). Low-dose corticosteroids (≤10 mg/day) could produce better results, even though the platelet counts are lower during the pregnancy period (40). Another study found that GC therapy of 1 mg/kg for ITP patients during pregnancy is less efficient than the non-pregnant population and increases the incidence of hypertensive disorders (41). A recent study showed that low doses of prednisone (5 mg once a day) and aspirin (75 mg once a day) were associated with a significant reduction in the titer of aβ2GPI, and women who successfully delivered exhibited significantly greater reductions of aβ2GPI than patients who experienced a fetal loss (42). In our study, the median daily dose of prednisone is 13.75 mg, which does not increase the risk of adverse pregnancy outcomes due to thrombocytopenia. These findings could suggest that lower starting doses of prednisone are appropriate in pregnant women with thrombocytopenia.

Our study has several limitations. First, it is a retrospective design, and we do not have complete information on all APS patients. Second, thrombocytopenia fluctuated in most patients, but we tried our best to record a similar time in the first trimester. Third, the sample size is relatively small, and therefore, we could not perform a stratified analysis. As such, we recommend conducting prospective cohort studies in a larger population.

## Conclusion

The study shows that thrombocytopenia in the first trimester increases the risk of premature birth at <37 weeks in OAPS patients. Effective treatments may improve pregnancy outcomes without increasing the risk of antepartum and postpartum hemorrhage. These results are important for improving the treatment of APS patients with thrombocytopenia.

## Data availability statement

The original contributions presented in the study are included in the article/**Supplementary Material**. Further inquiries can be directed to the corresponding authors.

## Ethics statement

The studies involving human participants were reviewed and approved by the ethics committee of Peking University People’s Hospital (2019PHB252). The patients/participants provided their written informed consent to participate in this study.

## Author contributions

JJ, XX, JL, ML, and CL conceived of and designed the project. LH and YH collected and input the clinical and laboratory data. JJ completed the statistical analyses. JJ, ML, and CL conducted the table and figure calculations. JJ and CL wrote the manuscript. All authors provided critical feedback and helped shape the research, analysis, and manuscript. All authors discussed the results and contributed to the final manuscript.

## Funding

This work was supported in part by China International Medical Foundation (No. Z-2018-40-2101), the National Natural Science Foundation of China (No.81871281), the Beijing Natural Science Foundation (7192211), and the Peking University People’s Hospital Research And Development Funds Project (RDY2021-10).

## Acknowledgments

We would like to thank all patients who participated in this study. We also thank Xingchen Li, Ph.D. of Peking University People’s Hospital, for helping with the statistics and with revising the manuscript.

## Conflict of interest

The authors declare that the research was conducted in the absence of any commercial or financial relationships that could be construed as a potential conflict of interest.

## Publisher’s note

All claims expressed in this article are solely those of the authors and do not necessarily represent those of their affiliated organizations, or those of the publisher, the editors and the reviewers. Any product that may be evaluated in this article, or claim that may be made by its manufacturer, is not guaranteed or endorsed by the publisher.
