# Supplementary material for: Thrombocytopenia in the first trimester predicts adverse pregnancy outcomes in obstetric antiphospholipid syndrome
Source: Front Immunol. 2022 Aug 18;13:971005. doi: 10.3389/fimmu.2022.971005 (PMC9433896; doi:10.3389/fimmu.2022.971005)
Supplement: Supplementary file 1 [file DataSheet_1.pdf]

Supplementary Material

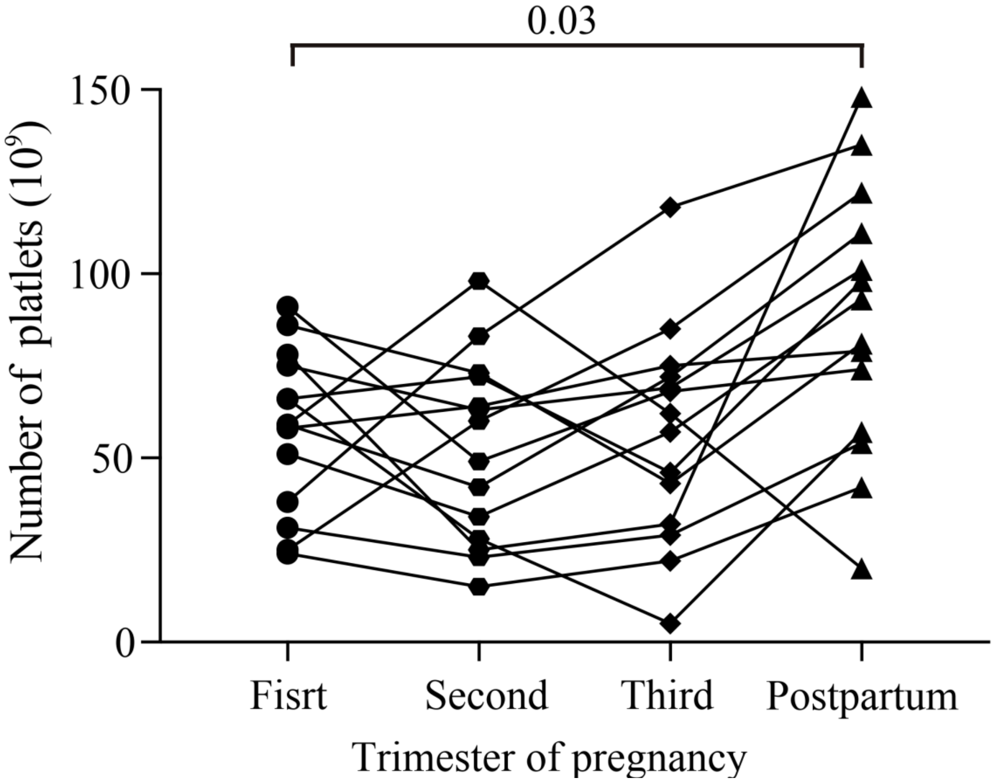

Supplementary Figure1. The number of platelets increased after delivery with the treatment in 14 thrombocytopenia patients with live fetus (57.64 ± 5.77 vs. 86.79 ± 9.65×10^9/L, p = 0.03).

Supplementary Table 1. The management and outcomes of patients with or without a history of thrombocytopenia in the thrombocytopenia group.

Thrombocytopenia history before pregnancy	No (N=2)	Yes (N=14)	*P*
Pred, mg/d	5.00 (2.50-7.50)	15.00 (10.00-18.75)	0.169
Pred, n (%)	1 (50.00)	11 (78.57)	0.450
HCQ, mg/d	400.00 (400.00-400.00)	300.00 (200.00-400.00)	0.215
HCQ, n (%)	2 (100.00)	11 (78.57)	0.999
Aspirin, mg/d	0.00 (0.00-0.00)	0.00 (0.00-37.50)	0.403
Aspirin, n (%)	0 (0.00)	4 (28.57)	0.999
LMWH, IU/d	0.00 (0.00-0.00)	0.00 (0.00-3075.00)	0.402
LMWH, n (%)	0 (0.00)	4 (28.57)	0.999
Aspirin+ LMWH, n (%)	0 (0.00)	1 (7.14)	0.999
IVIg, n (%)	1 (50.00)	3 (21.43)	0.450
SGA, n (%)	2 (100.00)	3 (21.43)	0.083

Premature birth <37 weeks, n (%)	2 (100.00)	5 (35.71)	0.175
Intrauterine fetal death, n (%)	2 (100.00)	0 (0.00)	0.008*
Stillbirth, n (%)	0 (0.00)	0 (0.00)	NA
PROM, n (%)	0 (0.00)	1 (7.14)	0.999
Preeclampsia, n (%)	0 (0.00)	1 (7.14)	0.999

* $P < 0.05$. Pred, prednisone; HCQ, hydroxychloroquine; LMWH: low molecular weight heparin, IVIg, Intravenous Immunoglobulin Therapy; SGA: small for gestational age; PROM: Premature rupture of membranes
